# Chimeric diphtheria toxin–CCL8 cytotoxic peptide for breast cancer management

**DOI:** 10.1002/1878-0261.70079

**Published:** 2025-06-22

**Authors:** Bernardo Chavez, Asieh Naderi, Kim‐Tuyen Huynh‐Dam, Vitali Sikirzhytski, Ioulia Chatzistamou, Hippokratis Kiaris

**Affiliations:** ^1^ Department of Drug Discovery and Biomedical Sciences College of Pharmacy, University of South Carolina Columbia SC USA; ^2^ Department of Pathology, Microbiology and Immunology School of Medicine, University of South Carolina Columbia SC USA; ^3^ Peromyscus Genetic Stock Center, University of South Carolina Columbia SC USA

**Keywords:** breast cancer, chemokine, cytotoxic peptide, toxin

## Abstract

Deregulation of chemokine CCL8 expression is common in various malignancies and other pathologies and plays a causative role in disease progression. However, despite CCL8's acknowledged role in pathology, inhibition of its activity does not represent a strategy of choice for cancer management. This is because its function overlaps with that of the other structurally related chemokines, and its activity is mediated by more than one receptor. To overcome this limitation, we hypothesized that ablation of CCL8 cellular targets, as opposed to disruption of CCL8 activity, may be more advantageous. Therefore, we developed DTCCL8, a chimeric cytotoxic peptide that delivers diphtheria toxin into cells expressing CCL8 receptors which are overexpressed in both the cells of tumor microenvironment and the cancer cells. The specificity of this peptide was confirmed *in vitro* by testing the cytotoxic activity of breast cancer cells overexpressing CCR5, a major CCL8 receptor, and by a neutralizing anti‐CCL8 antibody we developed. *In vivo*, DTCCL8 transiently reduced lymphocytes in blood, and its anticancer activity was confirmed in mouse breast cancers triggered by the polyoma middle T oncogene. These findings suggest that DTCCL8 can be used as a prototype for the development of a novel class of breast cancer therapeutics, targeting chemokine targets instead of inhibiting their activity. These cytotoxic peptides may also be useful for managing cancers and other immune system‐associated pathologies.

## Graphical abstract

DTCCL8 is a recombinant fusion toxin that targets cancer cells expressing chemokine receptors. By combining diphtheria toxin with CCL8, DTCCL8 binds to multiple receptors on tumor cells and induces selective cytotoxicity. This strategy enables receptor‐mediated targeting of cancer and may support the development of chemokine‐guided therapeutics.

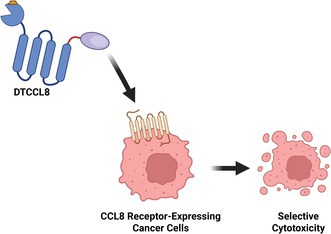

**Abbreviations**
ARDS: acute respiratory distress syndrome
DT: diphtheria toxin
DTT: dithiothreitol
GVHD: graft versus host disease
i.p: intraperitoneally
IPTG: isopropyl b‐D‐1‐thiogalactopyranoside
LYM: lymphocytes
MCP: monocyte chemotactic protein
MON: monocytes
NEU: neutrophils
PymT: polyoma middle T
scFV: single chain fragment variable
WBC: white blood cells

## 1 Introduction

Chemoattractive cytokines (chemokines) are essential for the development and progression of various cancers [1, 2, 3] Their activity targets both cancer cells and stromal cells including immune cells and fibroblasts, promoting cancer growth and metastatic spread, by producing antiapoptotic and mitogenic activity and by contributing to the transition of microenvironment from normal into pro‐oncogenic proinflammatory microenvironment. CCL8 in particular, or alternatively designated as monocyte chemotactic protein‐2 (MCP2), has been associated with the development of various cancers including breast cancer, by mechanisms involving the activation of tumor stroma and establishment of a gradient that favors dissemination of breast cancer cells, and the maintenance of a stem cell niche [4, 5, 6, 7, 8, 9, 10].

In addition to cancers, CCL8 has also been associated with various immune system‐related pathologies such as graft versus host disease (GVHD), microbial infections, and pulmonary fibrosis [11, 12, 13, 14, 15]. More recently, a role for CCL8 has been proposed for the development of acute respiratory distress syndrome (ARDS) after SARS‐CoV‐2 infection, while the beneficial effects of CCL8 inhibition have been described following lipopolysaccharide administration in mice [16, 17, 18, 19].

The evidence linking CCL8 with cancer development advocate for the development of CCL8‐based therapeutics to inhibit CCL8 activity. Nonetheless, no major attempts to develop CCL8‐targeting moieties exist so far, and only few efforts to target similar chemokines have been implemented, with limited success. The reason behind this gap is probably related to a combination of factors. For instance, CCL8 is highly similar to other chemokines of the same cytokine cluster, such as CCL7 and CCL11, exhibiting redundancy in their activities [4, 20]. Thus, inhibition of CCL8 would be inadequate in effectively suppressing the corresponding activities that lead to pathology. In addition, each of these chemokines recognizes more than one receptor. Additionally, CCL8 is an agonist for C‐C chemokine receptor type 2 (CCR2), CCR3, CCR5; therefore, targeting a single receptor instead of the ligand would be insufficient for the prevention of the corresponding activity [21, 22, 23, 24].

To overcome these limitations, we hypothesized that instead of targeting either CCL8 or its receptors, a strategy of choice would be to target the cellular targets of CCL8, irrespective of the specific receptor types that are expressed in the corresponding cell types. The premise of this hypothesis is that the receptors for CCL8, especially CCR2 and CCR5, are overexpressed in breast cancers, and thus, their presence sensitizes cells against specific receptor‐targeting moieties [25, 26, 27, 28, 29]. In addition, responsiveness to CCL8 is a feature of the tumor microenvironment, expanding the roster of the pro‐oncogenic targets in tumors [4, 5, 6]. To test this hypothesis, we developed the DTCCL8 prototype, which is a chimeric peptide that consists of CCL8 linked to the highly cytotoxic diphtheria toxin (DT). Similar strategies have been employed for the management of different cancers with encouraging results. In most cases, the cytotoxic peptide consisted of DT conjugated to IL‐2, IL‐3, or the single‐chain fragment variable (scFV) against the CD19 antigen [30, 31, 32, 33] and in the case of the IL‐3 conjugate, the corresponding drug, designated *Tagraxofusp*, has received approval for the treatment of hematologic malignancies [34]. Here, we show that DTCCL8 effectively targets cells in a manner that depends on CCR5 expression and that its activity is antagonized by a neutralizing anti‐CCL8 antibody. *In vivo*, DTCCL8 inhibited the growth of mouse breast cancers that were induced by the polyoma middle T (PymT) oncogene.

## 2 Materials and methods

### 2.1 Design and construction of fusion toxin vectors

A custom plasmid containing the human CCL8 fusion toxin construct (DT386‐hCCL8‐6xHis) was synthesized by GenScript and cloned into the pET30a(+) backbone between the NdeI and HindIII restriction sites. The DT386 portion includes the first 386 amino acids of the diphtheria toxin. DT386 and hCCL8 are connected by a (G_4_S)_2_ (four glycines and one serine residue linker). Six histidines (6xHis tag) were added at the N‐terminal for purification purposes. The sequence of the construct was verified by Sanger sequencing. To produce a truncated diphtheria toxin, we used splicing overlap extension polymerase chain reaction (SOEingPCR) with the following primers: Forward, 5′‐CATATGCACCATCACCACCA containing NdeI cut site and reverse, 5′‐AAGCTTTTAGCCGGTTTTATGACC containing HindIII cut site. This fragment was then cloned into a second plasmid, from which the original construct was removed by digestion with NdeI and HindIII. The new construct containing only the DT386 fragment was then cloned into the pET30a(+) backbone using these restriction sites, yielding a truncated diphtheria construct.

### 2.2 Expression and purification of fusion toxins

Both plasmids were transformed into BL21 (DE3) *E. coli* cells independently. Positive clones were observed in LB (Luria‐Bertani) agar plates with 50 μg·mL^−1^ kanamycin. A single colony was picked and transferred to 4 mL LB media with 50 μg·mL^−1^ kanamycin. The starter culture was grown at 37 °C overnight at 250 r.p.m. The starter culture was then expanded in 1 L of LB (Luria‐Bertani) medium (supplemented with kanamycin at 50 μg·mL^−1^) by incubating at 37 °C and 250 r.p.m. until the OD_600_ reached 0.7. Protein expression was induced with 0.3 mm isopropyl b‐D‐1‐thiogalactopyranoside (IPTG) and further cultured for 3 h at 37 °C. Cells were harvested by centrifugation, and the expression of DT386‐CCL8 in total cell protein from uninduced and induced samples was analyzed by SDS/polyacrylamide gel electrophoresis (SDS/PAGE). Cell pellet was lysed using B‐PER Bacterial Protein Extraction Reagent (Pierce), 0.2 mg·mL^−1^ lysozyme (ThermoFisher Scientific, Waltham, MA, USA), 1× Halt Protease Inhibitor Cocktail (ThermoFisher Scientific), and DNase I (Invitrogen). Inclusion bodies were dissolved using Inclusion Body Solubilization Reagent (Pierce) and 1 mm dithiothreitol (DTT). The protein refolding method was adapted from the protocol described by [35]. Briefly, the solubilized inclusion bodies were added to a refolding buffer containing 50 mm Tris/HCl, pH 8.5, 0.4 m sucrose, 10% glycerol, 0.5% Triton X‐100, 0.3 mm glutathione disulfide (GSSG), and 3 mm glutathione (GSH) at a rate of 500 μL·h^−1^ while being stirred at 200 r.p.m. at 4 °C. The refolding solution was then stirred for 72 h at 4 °C. The solution was centrifuged at 9000 ***g*** for 20 min at 4 °C, and the supernatant was collected for purification. The His‐tagged proteins were purified using Ni‐NTA affinity chromatography independently. The supernatant fraction of the refolded protein was supplemented with sodium chloride to a final concentration of 1.5 m and imidazole to 5 mm. The refolded solution was then applied to Ni‐NTA resin (GenScript) pre‐equilibrated with binding buffer (50 mm Tris/HCl, pH 8.0, 300 mm NaCl, 10 mm imidazole). After incubation for 30 min at 4 °C, the resin was washed with 20 column volumes of wash buffer (50 mm Tris/HCl, pH 8.0, 300 mm NaCl, 20 mm imidazole). The bound protein was eluted with elution buffer (50 mm Tris/HCl, pH 8.0, 300 mm NaCl, 250 mm imidazole).

### 2.3 SDS/PAGE and western blot

The purity and identity of the protein were assessed by SDS/PAGE and western blot analysis. For SDS/PAGE, protein samples were mixed with Laemmli buffer and heated at 95 °C for 5 min before loading onto a 12% polyacrylamide gel. Following electrophoresis, the gel was stained with Coomassie Brilliant Blue to visualize protein bands. The fractions containing the desired protein were pooled and subjected to buffer exchange into PBS (pH 7.4) using Pierce Protein Concentrators PES (Pierce) with a 10 K molecular weight cut‐off (MWCO). Following buffer exchange, the pooled protein fractions were processed to remove endotoxins using Pierce High‐Capacity Endotoxin Removal Spin Columns (Pierce) according to the manufacturer's instructions. The protein concentration was then determined using a BCA assay. For western blotting, proteins were transferred from the gel onto a PVDF membrane. Two separate western blots were performed. The first blot was probed with Mouse6xHis Tag mAb HRP (MA1‐21315‐HRP) to confirm the presence of the His‐tagged protein. The second blot was probed with a chimeric antibody targeting human CCL8, followed by a mouse anti‐Human IgG1 Fc Secondary Antibody, HRP (a10648). Detection was carried out using an enhanced chemiluminescence (ECL) substrate.

### 2.4 Cell culture

HEK293T (RRID:CVCL_0045), MDA‐MB‐231 (RRID:CVCL_0062, TNBC), BT‐474 (RRID:CVCL_0179, HER2+, ER+, PR+), BT‐549 (RRID:CVCL_1092, TNBC), and MCF‐7 (RRID:CVCL 0031, HER2‐, ER+, PR+) cells were cultured in Dulbecco's modified Eagle medium (DMEM; Gibco, Thermofisher Scientific, Waltham, MA, USA) supplemented with 10% fetal bovine serum (FBS; Gibco) and 1% penicillin–streptomycin (Pen/Strep; Gibco). CHO‐K1 cells were cultured in Ham's F‐12 Nutrient Mixture (F‐12; Gibco) with the same supplements. Cell lines were originally obtained by ATCC and thereafter maintained in our laboratory. All cell lines were maintained at 37 °C in a humidified atmosphere with 5% CO_2_. All experiments were performed with mycoplasma‐free cells. Mycoplasma was tested by using the MycoAlert^®^ Mycoplasma Detection Kit (Lonza, Walkersville, MD, USA). Human cell lines have been validated by standard STR analysis in the past three years by the Functional Genomics Core of the Center for Targeted Therapeutics.

### 2.5 Cytotoxicity studies with DTCCL8 and DT

To evaluate the cytotoxicity of DTCCL8 and DT, *in vitro* experiments were conducted using CHO‐K1, MDA‐MB‐231, MCF‐7, BT‐474, BT‐549, and HEK293T cell lines. Cells were seeded in 96‐well plates at a density of 7000 cells per well in triplicate. The cells were allowed to adhere overnight and then treated with a series of nine concentrations of each toxin variant, starting at 2500 nm and using serial dilutions to achieve final concentrations of 1250, 625, 312.3, 156.3, 78.1, 39, 19.5, and 9.7 nm. The treatments were applied for 72 h. Following the incubation period, cell viability was assessed using the XTT assay. The XTT reagent was added to each well according to the manufacturer's instructions, and the plates were incubated for an additional 4 h at 37 °C. The absorbance was then measured at 450 nm with a reference wavelength of 650 nm using a microplate reader.

### 2.6 Transient transfection with human CCR5 receptor

CHO‐K1 and MDA‐MB‐231 cells were transiently transfected with a plasmid encoding the human CCR5 receptor (pcDNA3‐CCR5, a gift from Erik Procko; Addgene plasmid # 98943; http://n2t.net/addgene:98943; RRID:Addgene_98 943). Cells were seeded in 96‐well plates at a density of 7000 cells per well in triplicate and allowed to adhere overnight. The next day, transfection complexes were prepared using Lipofectamine 3000 (Invitrogen) according to the manufacturer's protocol. For each well, the CCR5 plasmid DNA was mixed with Lipofectamine 3000 reagent in Opti‐MEM (Gibco) and incubated for 15 min at room temperature to form DNA–Lipofectamine complexes. The transfection complexes were then added to the cells, and the plates were incubated at 37 °C in a 5% CO_2_ atmosphere for 24 h to allow for transgene expression. After the transfection period, cells were treated with varying concentrations of the toxin for 72 h. Following the treatment, cell viability was assessed using the XTT assay.

### 2.7 CCR5 blockade with neutralizing antibody

MDA‐MB‐231 and BT549 cells were seeded at 7000 cells per well in 96‐well plates (triplicates per condition) and allowed to adhere overnight. Cells were pre‐treated with a CCR5‐blocking antibody (clone 45 531; R&D Systems, Minneapolis, MN, USA) at 10 μg·mL^−1^ for 1 h prior to toxin exposure. Following pre‐treatment, cells were treated with 500 nm of DTCCL8 or DT386 while maintaining the antibody in the media at a concentration of 7.5 μg·mL^−1^ throughout the 7‐day experiment. Control wells included untreated cells, cells treated with antibody alone, and cells treated with toxin without antibody. Media containing antibody and toxin was replenished on day 3. On day 7, cell viability was quantified using the XTT assay. Results were normalized to untreated controls.

### 2.8 Fluorescent labeling and internalization assay

DTCCL8 and DT386 were fluorescently labeled using DyLight™ 633 NHS Ester (Thermo Fisher Scientific, Waltham, MA, USA), which covalently binds to primary amines (typically lysine residues) on the protein. The labeling reactions were carried out according to the manufacturer's protocol, and Pierce™ Dye Removal Columns (Thermo Fisher Scientific) were used to eliminate excess unbound dye. MDA‐MB‐231 and BT549 cells were seeded in 96‐well glass‐bottom plates (P96‐1.5H‐N; Cellvis, Mountain View, CA, USA) pre‐coated with Poly‐D‐Lysine (A3890401; Thermo Fisher Scientific) at a density of 10 000 cells per well in triplicate and incubated overnight. Cells were treated for 24 h with 50 nm or 25 nm of DyLight 633‐labeled DTCCL8 or DT386. For competition conditions, the labeled DTCCL8 peptide was co‐incubated with a tenfold molar excess of either a chimeric anti‐CCL8 antibody or a human IgG1 isotype control (500 nm or 250 nm, respectively). Imaging was performed using a Zeiss LSM 700 confocal microscope equipped with a 10× objective. For each well, three representative fields were captured. Fluorescence intensity was quantified using ImageJ by measuring the total cell intensity around automatically detected nuclei using a custom thresholding algorithm, with manual validation of nuclei segmentation quality. Quantified values were normalized to control‐treated wells.

### 2.9 Tumor growth studies

Mice were originally obtained from Jackson Labs and subsequently maintained in our facilities at 12 h light/12 h dark cycle, under standard chow diet provided *ad libitum*. Wild‐type female C57BL/6 mice, aged 2–4 months, were used for the *in vivo* analysis. Mammary tumors from B6.FVB‐Tg(MMTV‐PyMT; JAX stock # 022974, [36]) mice backcrossed into C57BL/6 background were harvested and implanted into the flanks of 21 recipient mice using a trocar needle. A single tumor was used for all implantations to ensure consistency. Tumor growth was monitored, and once the tumors reached a similar size across all mice, the animals were randomly assigned into two groups: a control group (*n* = 10) and a treatment group (*n* = 11). The treatment group received 1.5 mg·kg^−1^ of DTCCL8 administered intraperitoneally (i.p.) every two days, while the control group received an equivalent volume of vehicle solution. Tumor sizes measured using calipers were recorded twice a week. The volume of the tumors was calculated using the formula: *V* = 0.5 × *L* × *W* ^2^. Mice were observed for signs of distress or adverse reactions throughout the study period. Tumor size and weight were meticulously recorded to assess the efficacy of the DTCCL8 treatment compared to the control.

### 2.10 Blood collection and analysis

Blood samples were collected from female C57BL/6 mice aged 2–4 months at specified time points for two treatment groups. For the first group, blood was collected 5 days before treatment, and 1, 6, and 11 days after treatment with DTCCL8 at a dosage of 1.5 mg·kg^−1^. Blood was drawn from the submandibular vein using a sterile lancet and collected into EDTA‐coated microcentrifuge tubes to prevent clotting. On each collection day, mice were gently restrained, and the puncture site was cleaned with an alcohol swab. Using a sterile lancet, a small puncture was made in the submandibular vein and approximately 50–100 μL of blood was collected per mouse. After blood collection, gentle pressure was applied to the puncture site with sterile gauze to stop any bleeding. The collected blood samples were gently mixed by inversion to ensure proper anticoagulation. Hematological parameters were measured using the Vetscan HM5 hematology analyzer (Abaxis), following the manufacturer's instructions.

All procedures involving animals were conducted in accordance with the ethical guidelines and standards set forth by the Institutional Animal Care and Use Committee (IACUC) at the University of South Carolina. The study was approved by the IACUC of the University of South Carolina under protocol number 2614‐101747‐082922.

## 3 Results

### 3.1 Development of DTCCL8

**Fig. 1:**
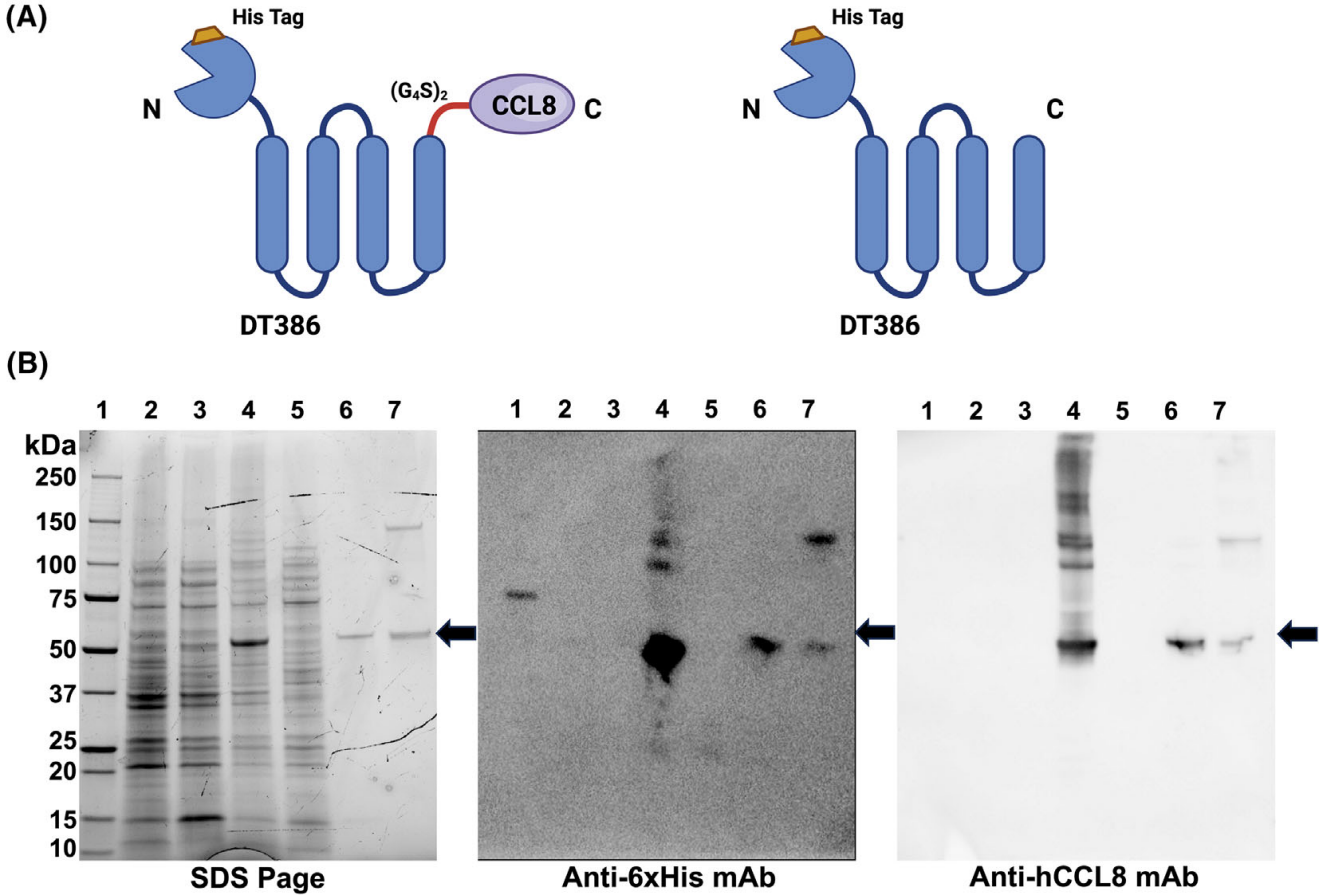
Structure and expression of DTCCL8. (A) Schematic illustration of the components of the conjugate DTCCL8 (DT386‐CCL8, left) or DT386 (right) is indicated. DTCCL8 is composed of the truncated diphtheria toxin (DT386) fused with the human chemokine CCL8. (B) SDS/PAGE and western blot characterization of purified DTCCL8 expressed in *E. coli*. SDS/PAGE analysis (left), western blot analysis using anti‐6xHis monoclonal antibody (mAb) (middle), and western blot analysis using anti‐human CCL8 mAb (right) of untransformed BL21 (lane 2), transformed BL21 with pET30a‐DT386‐CCL8, uninduced (lane 3), transformed BL21 with pET30a‐DT386‐CCL8 induced with 0.3 mM IPTG (lane 4), soluble proteins from lane 4 (lane 5), inclusion bodies isolated from lane 4 (lane 6), refolded and purified DTCCL8 via Nickel column chromatography. Arrow indicates the predicted ~ 52.5 kDa band. Lane 1, molecular weight marker.

Using recombinant DNA technology, we generated a pET30a‐based plasmid expressing a chimeric peptide consisting of diphtheria toxin (DT386) and the full‐length human CCL8, separated by a dimer of 4XGlycine‐Ser linker (Fig. 1A and Fig. S1). A His tag was introduced in the N terminus of the DT386 to enable identification. As shown in Fig. 1B and Figs. S2 and S3, the purified peptide (DTCCL8) had the expected size of about 52.5 kDa and was detectable using both anti‐His and CCL8‐specific antibody that we previously developed [19]. Under reducing conditions, a single band was seen (Fig. 1B, lane 6) while following refolding, a larger band, likely corresponding to a DTCCL8 multimer, appeared (Fig. 1B, lane 7). Further characterization of DT386 expression and purification, including SDS/PAGE profiles and analysis under reducing and non‐reducing conditions, is shown in Figs S2 and S3.

### 3.2 DTCCL8 activity is sensitized by CCR5 expression and is antagonized by anti‐CCL8 treatment

**Fig. 2:**
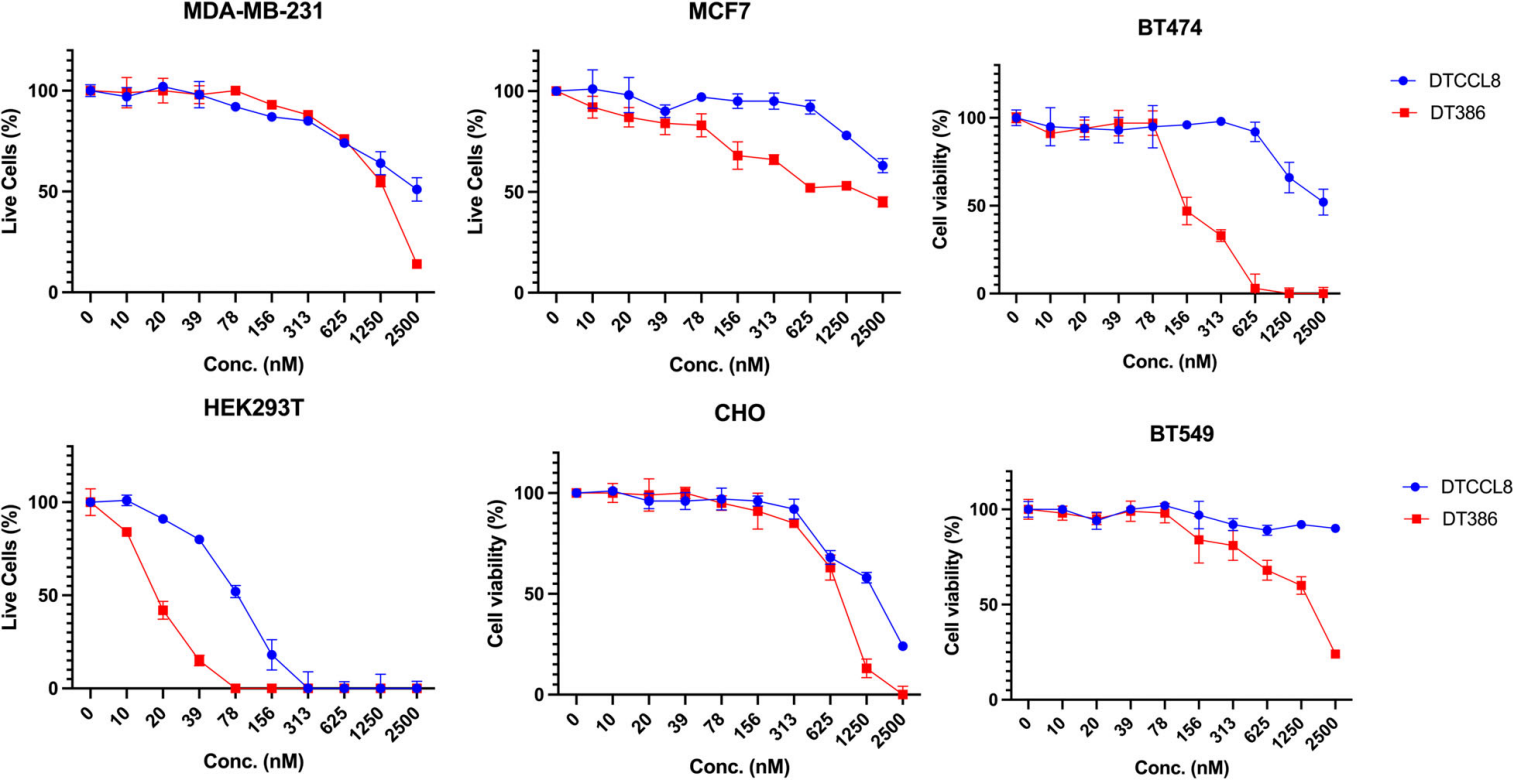
Cytotoxicity of DTCCL8 compared to unconjugated DT386 across multiple cell lines. Human (MDA‐MB‐231, MCF7, BT474, HEK293T, BT549) and non‐human (CHO) cell lines were treated with increasing concentrations of DTCCL8 or DT386 for 3 days. Cell viability was measured and is shown as a percentage of untreated controls. DT386 consistently induced greater cytotoxicity than DTCCL8 across all tested lines. BT549 cells appeared resistant to DTCCL8 at 3 days but showed sensitivity after 6 days of treatment (Fig. S4). Error bars represent mean ± SEM. *n* = 3 independent experiments performed in triplicate.

Initially, we confirmed that DTCCL8 can be internalized by cells using time‐lapse confocal imaging of a HEK293T cell treated for 24 h with fluorescently labeled DTCCL8 (Video S1) Then, we assessed the cytotoxicity of DTCCL8 in comparison with an equimolar amount of unconjugated DT386 toxin in different cancer cell lines. DTCCL8 was consistently slightly less toxic than DT386 at equimolar concentrations (Fig. 2, Fig. S4). While DTCCL8 is designed for receptor‐mediated targeting, its reduced toxicity at high concentrations may reflect variable or low expression of CCL8 receptors in monoculture or potential receptor saturation. In contrast, DT386 may enter cells through receptor‐independent mechanisms, particularly at high concentrations, as previously reported for truncated diphtheria toxins [37]. Consistent with this, DT386 administered at 1.5 mg·kg^−1^ in mice was acutely toxic, leading to death in treated animals, whereas DTCCL8 at the same dose was well tolerated and used in subsequent *in vivo* studies (see below).

**Fig. 3:**
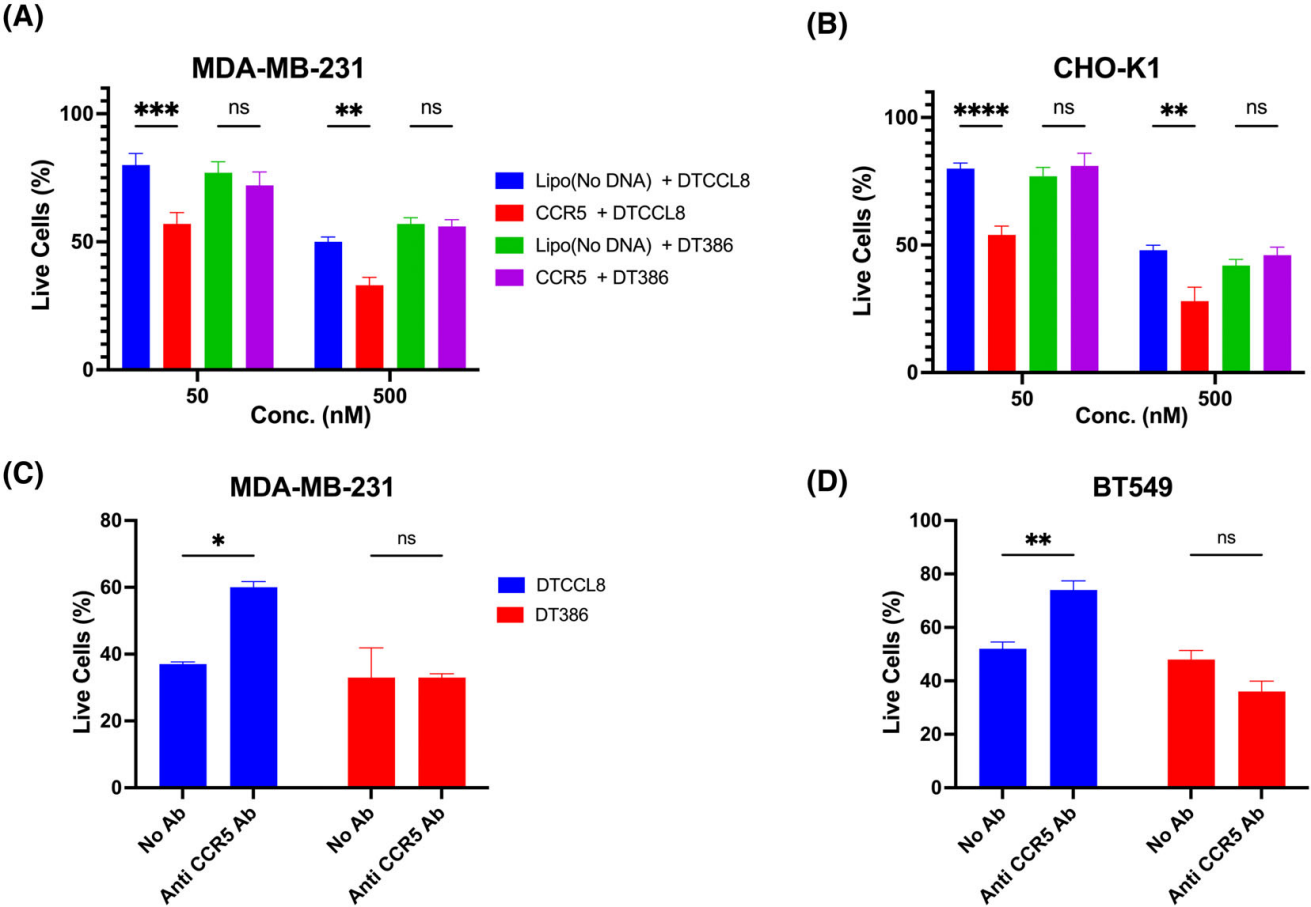
DTCCL8 cytotoxicity depends on CCR5 expression. (A) MDA‐MB‐23cells transfected with CCR5 show increased sensitivity to DTCCL8 at concentrations (conc.) of 50 or 500 nM compared to control cells. (B) CHO‐K1 cells transfected with CCR5 also display enhanced DTCCL8‐induced cytotoxicity. (C) In MDA‐MB‐231 cells, CCR5 blockade using an inhibitory anti‐CCR5 antibody (clone 45 531) reduces DTCCL8‐induced cytotoxicity. (D) A similar protective effect is observed in BT549 cells treated with anti‐CCR5 antibody. For transfection experiments (A and B), cells were treated with DTCCL8 or DT386 for 3 days following CCR5 transfection. For antibody blockade experiments (C and D), cells were pre‐treated with anti‐CCR5 antibody (10 μg·mL^−1^) for 1 h before toxin exposure and maintained in 7.5 μg·mL^−1^ antibody throughout the 7‐day treatment period. Error bars represent mean ± SEM. Statistical significance was determined using two‐way ANOVA with Sidak's multiple comparisons test. *P < 0.05, **P < 0.01, ***P < 0.001, ****P < 0.0001, ns = not significant. *n* = 3 independent experiments performed in triplicate.

Next, we transiently overexpressed CCR5, the major CCL8 receptor, in MDA‐MB‐231 and CHO‐K1 cells and explored the toxicity of DTCCL8 and DT386. As shown in Fig. 3A,B, expression of CCR5 sensitized both cell types against DTCCL8, conferring cytotoxicity at concentrations at which untransfected cells are resistant to the drug. In contrast, DT386 toxicity was unaffected by CCR5 expression, indicating that DT386 does not benefit from CCR5‐mediated uptake. These results confirm that DTCCL8 cytotoxicity is mediated specifically through CCL8 receptor engagement. To assess the role of CCR5 in DTCCL8 activity, MDA‐MB‐231 and BT549 cells were pre‐treated with a CCR5‐blocking antibody (clone 45 531) prior to exposure to 500 nm DTCCL8 or DT386. The antibody was maintained throughout the 7‐day treatment period. DTCCL8‐induced cytotoxicity was reduced in both cell lines following CCR5 blockade, while DT386 toxicity was unaffected (Fig. 3C,D).

**Fig. 4:**
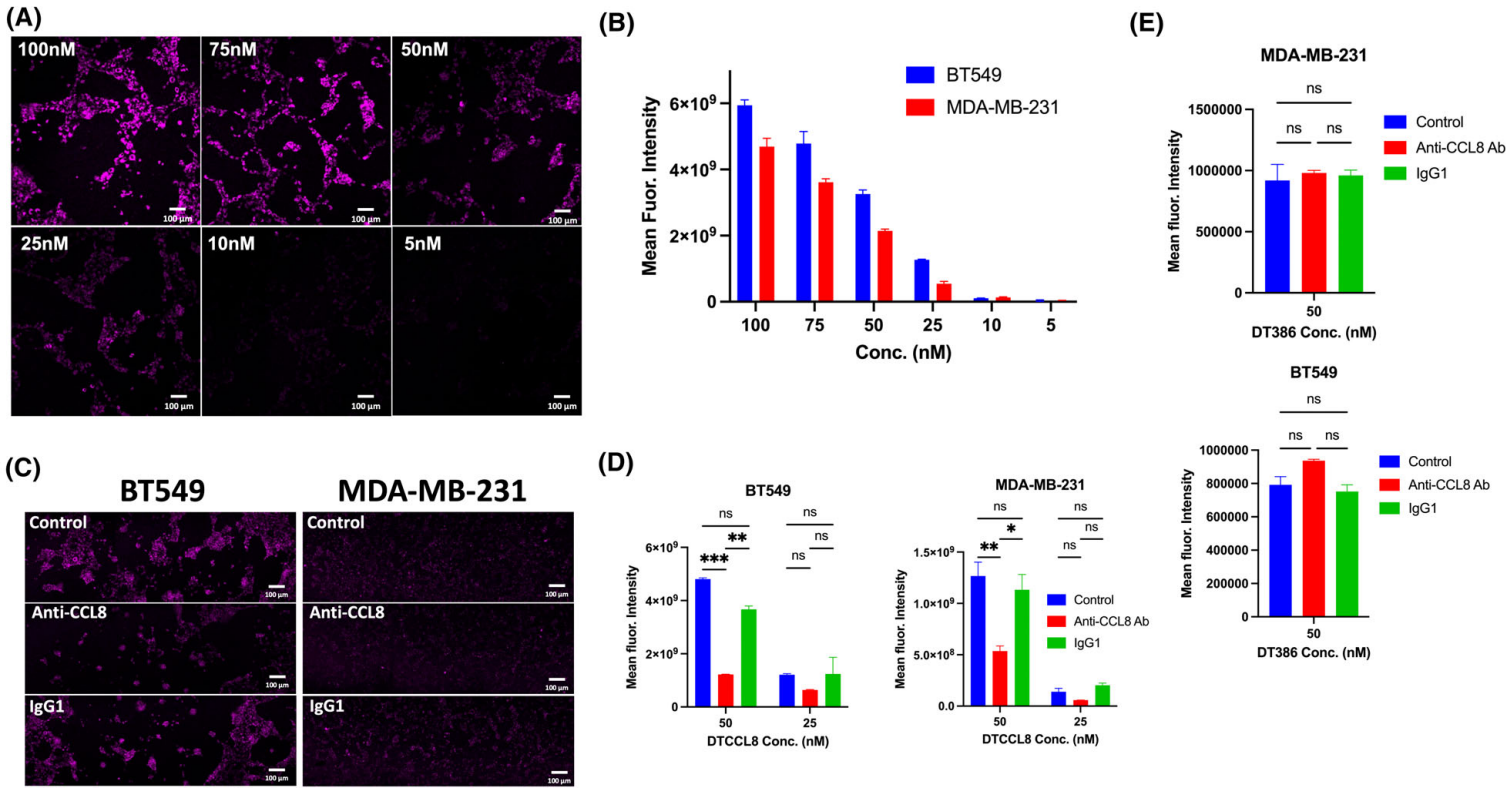
Internalization of DTCCL8 by breast cancer cells. (A) Confocal microscopy images of BT549 cells treated with fluorescently labeled DTCCL8 (magenta) at concentrations (conc.) ranging from 5 to 100 nm. (B) Quantification of mean fluorescence intensity in BT549 and MDA‐MB‐231 cells following treatment with fluorescent DTCCL8 across a range of concentrations. (C) Representative confocal images showing that neutralization of CCL8 using anti‐CCL8 antibody reduces DTCCL8 internalization in BT549 and MDA‐MB‐231 cells compared to control or IgG1 treated cells. (D) Quantification of fluorescence intensity from panel C in BT549 and MDA‐MB‐231 cells treated with DTCCL8 (25 or 50 nm) with or without anti‐CCL8 antibody. (E) Quantification of fluorescence intensity in BT549 and MDA‐MB‐231 cells treated with DT386 (50 nm), with or without anti‐CCL8 antibody or IgG1 control antibody. Antibodies were used at a 10‐fold molar excess relative to the toxin in panels C–E. Scale bars: 100 μm. Error bars represent mean ± SEM. Statistical significance for panels D and E was determined using two‐way ANOVA with Sidak's multiple comparisons test. *P < 0.05, **P < 0.01, ***P < 0.001, ns = not significant. *n* = 3 independent experiments performed in triplicate.

To evaluate receptor‐mediated uptake, we assessed internalization of fluorescently labeled DTCCL8 and DT386 in breast cancer cells. Confocal imaging of BT549 cells treated with increasing concentrations of DTCCL8 revealed dose‐dependent internalization (Fig. 4A), and quantification confirmed concentration‐dependent uptake in both BT549 and MDA‐MB‐231 cells (Fig. 4B). To further assess the role of CCL8 receptor binding, cells were co‐treated with a neutralizing anti‐CCL8 antibody. DTCCL8 uptake was significantly reduced in the presence of the blocking antibody, while control IgG1 treatment had no effect (Fig. 4C,D). Internalization of DT386 was unaffected by either antibody treatment (Fig. 4E).

### 3.3 Effect of DTCCL8 on white blood cells (WBC) in mice

**Fig. 5:**
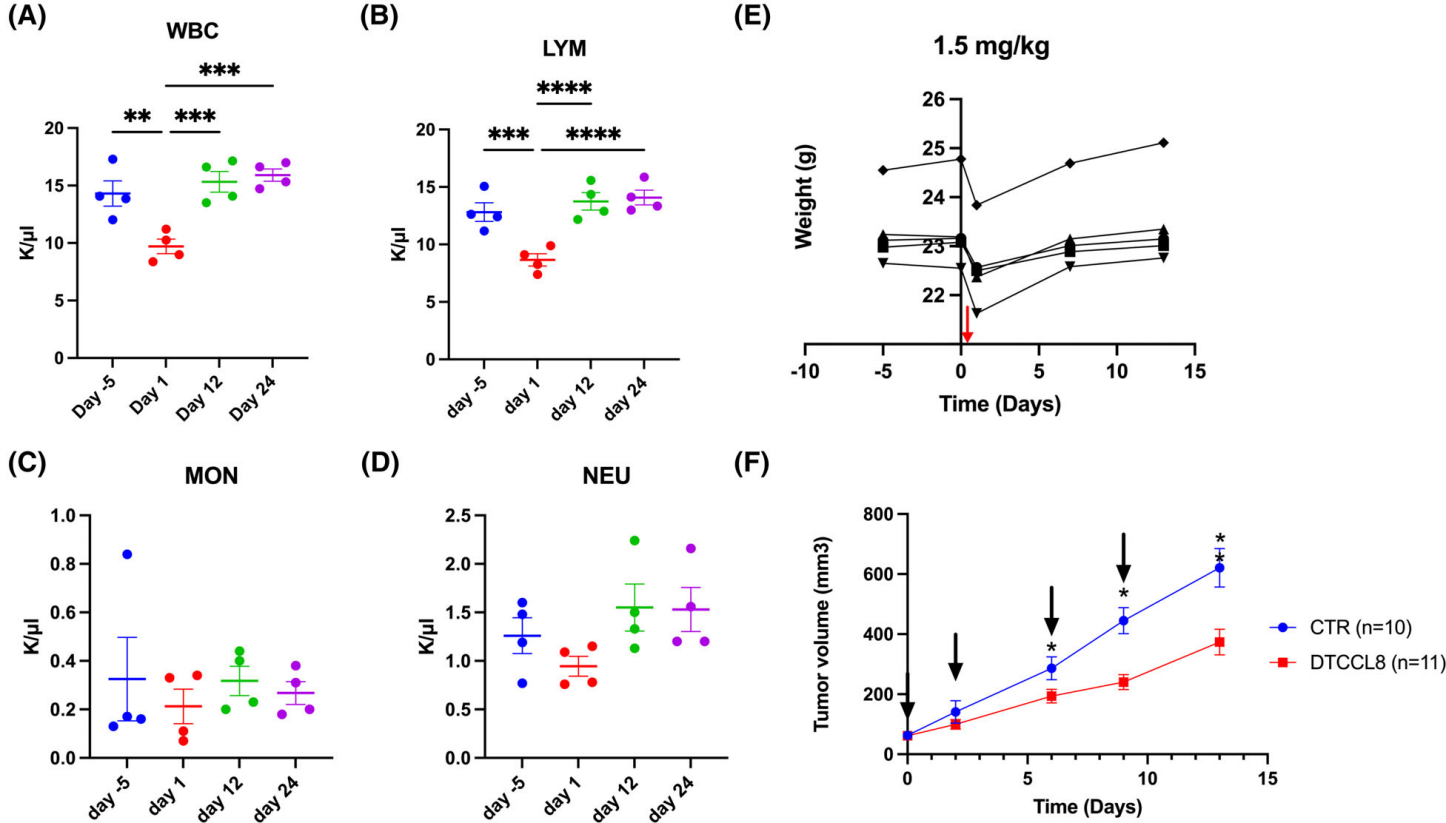
Effect of DTCCL8 on immune cell populations, body weight, and tumor growth. (A–D) Blood counts showing total white blood cells (WBC), lymphocytes (LYM), monocytes (MON), and neutrophils (NEU) following intraperitoneal administration of DTCCL8 (1.5 mg·kg^−1^ at day 0). (E) Body weight of mice (*n* = 5) treated with DTCCL8. Each line represents an individual mouse. Red arrow indicates the time of injection (F) Tumor volume in wild‐type mice bearing PyMT breast cancer isografts treated with DTCCL8 or vehicle. DTCCL8 was administered at 1.5 mg·kg^−1^ i.p. at the time points indicated by black arrows. CTR = control group (*n* = 10), DTCCL8 group (*n* = 11). Error bars represent mean ± SEM. Statistical significance was determined using two‐way ANOVA with Sidak's multiple comparisons test (A–D), and Student's *t*‐test (F). *P < 0.05, **P < 0.01, ***P < 0.001, ****P < 0.0001, ns = not significant.

Immune cells are responsive to CCL8 because they express several receptors for CCL8 such as CCR2, CCR3, CCR5, and others, and thus, they are predicted to be sensitive to DTCCL8. Therefore, we explored the effects of DTCCCL8 in the blood profile of wild‐type mice. To that end, mice received 1.5 mg·kg^−1^ DTCCL8 i.p. and their WBCs were assessed 5 days prior and 1, 6, and 11 days after the administration of the cytotoxic compound. The dose of DTCCL8 was determined by pilot experiments and was determined to be well tolerated. As shown in Fig. 5A–D, one day after the administration of DTCCL8, total white blood cell count was significantly decreased (Fig. 5A), but rebounded after 12 days. This decrease was primarily due to a decrease in lymphocytes (Fig. 5B) which are known to express CCR5 [38]. The effect of CCL8 in monocytes and neutrophils (Fig. 5C,D) was insignificant at this dose [38]. Body weight changes were insignificant after DTCCL8 administration (Fig. 5E). To confirm these findings, the treatment experiment was repeated independently, and a similar trend in WBC, lymphocyte, monocyte, and neutrophil counts was observed (Fig. S5).

### 3.4 DTCCL8 inhibits breast cancer growth in mice

Subsequently, we sought to evaluate the potential anticancer activity of DTCCL8 in breast cancers at which the effect of CCL8 is established. Since both cancer cells and cells of the microenvironment are responsive to CCL8, we sought to explore the effects of DTCCL8 in mouse breast cancer in mice that possess intact immune system, as opposed to human breast cancer xenografts at which the host's immune system is compromised. To that end, we utilized isografts of primary mouse breast cancers triggered by polyoma middle T oncogene, that were transplanted in wild‐type isogenic mouse hosts. These are highly aggressive tumors that double in size every 4–5 days. Mice were treated when tumors became measurable, at about 50–70 mm^3^. Treatment involved i.p. administration of DTCCL8 at 1.5 mg·kg^−1^ at days 0, 6, and 9, that is well tolerated by the mice. As shown in Fig. 5F, 9 days after initiation of therapy, tumor size decreased significantly in the DTCCL8 group compared to controls.

## 4 Discussion

We report the development and preliminary *in vitro* and *in vivo* characterization of DTCCL8, a cytotoxic peptide conjugate consisting of diphtheria toxin (DT) linked to the chemokine hCCL8. The development of this conjugate was prompted by the observation that CCL8 receptors, like other chemokine receptors, are overexpressed in several cancers including breast cancers [7]. However, their redundancy in expression profile, ligand specificity, and function reduces their potential utility in clinical practice [39]. The same redundancy applies not only to the receptors but to their ligands as well, for which neutralization of a single target would likely not provide clinically relevant results. Furthermore, the expression of these chemokine receptors is not limited to the cancer cells but to the cells of the tumor stroma, providing a promising therapeutic target [40].

We hypothesized that this redundancy in the expression of ligands and receptors, instead of a limitation, could be converted to an advantage, enabling targeting of cytotoxic agents to tumors irrespectively of the specific receptor signature, for as long as responsiveness to CCL8 is retained. To that end, we developed DTCCL8, which exhibits specific toxicity to cancer cells in a manner that depends on CCR5 and likely other CCL8 receptors' expression. This conclusion is further supported by our observation that antibody‐mediated CCR5 blockade significantly reduced DTCCL8‐induced cytotoxicity in both MDA‐MB‐231 and BT549 cells, while DT386 activity remained unaffected. These results confirm that DTCCL8 selectively targets cells through receptor engagement rather than nonspecific uptake. The cytotoxicity of DT386 was likely due to its internalization via non‐canonical mechanisms, as previously demonstrated for the structurally similar DT385 [37]. DTCCL8 was effective in inhibiting the growth of highly aggressive mouse breast cancer isografts, triggered by the middle T oncogene of polyoma virus. DTCCL8, at a dose that was effective against breast cancers, was not toxic in mice. As expected, transiently, a reduction of WBCs, due to lymphocyte decrease, was noted after DTCCL8 administration, but the effect was subsequently abolished.

The specific targeting of immune cells suggests that DTCCL8, besides cancers that are responsive to CCL8, may also be effective against other conditions that involve aberrant immune response activation. These include ARDS and GVHD, at which a massive accumulation of immune cells occurs, and both have been linked to supraphysiological levels of CCL8 expression [11, 13, 17, 18].

## 5 Conclusions

DTCCL8 represents a first of its class anticancer agent that targets tumors based on their capacity to respond to a specific chemokine such as CCL8. It is plausible that modulation, namely upregulation of CCL8 activity, in combination with other chemokine–cytotoxic drug combinations, may provide a novel strategy for the management of breast and other cancers, depending on their specific responsiveness to specific chemokines. It is noted that the potential availability of such cytotoxic peptides, in combination with the ability to rapidly evaluate the responsiveness of primary tumors to these drugs by *ex vivo* screening, will provide personalized strategies for the management of cancers exhibiting high deregulation in chemokine networks.

## Conflict of interest

The University of South Carolina has filed a patent application for DTCCL8 (Application No. 63/660798). BC and HK are designated as inventors in the application.

## Author contributions

BC designed and purified peptides. BC, AN, K‐T HD, and VS performed experiments. IC performed histopathology. BC and HK analyzed data and wrote the manuscript draft. HK and BC designed the study. HK supervised the study and acquired funding. All authors approved the manuscript.

## Peer review

The peer review history for this article is available at https://www.webofscience.com/api/gateway/wos/peer‐review/10.1002/1878‐0261.70079.

## Supporting information


**Fig. S1.** Annotated nucleotide and amino acid sequences of the DTCCL8 fusion construct including DT386, (G_4_S)_2_ linker, and N‐terminal 6xHis tag.
**Fig. S2.** SDS/PAGE analysis of DT386 expression and purification in *E. coli*.
**Fig. S3.** SDS/PAGE analysis of DTCCL8 and DT386 proteins under reducing and non‐reducing conditions.
**Fig. S4.** Cytotoxicity of DTCCL8 and unconjugated DT386 in BT549 and MDA‐MB‐231 cells.
**Fig. S5.** Effect of DTCCL8 on immune cell populations and body weight in an independent *in vivo* study.

**Video S1.** Time‐lapse confocal imaging of DTCCL8 uptake in a representative HEK293T cell.

## Data Availability

All data are shown in the manuscript and the accompanying Supporting Information. Peptide sequences are provided in Supporting Information. Peptides at small amounts are available from the corresponding author upon request.
