# Supplementary material for: Chimeric diphtheria toxin–CCL8 cytotoxic peptide for breast cancer management
Source: Mol Oncol. 2025 Jun 22;19(11):3376–86. doi: 10.1002/1878-0261.70079 (PMC12591328; doi:10.1002/1878-0261.70079)
Supplement: Supplementary file 1 — Fig. S1. Annotated nucleotide and amino acid sequences of the DTCCL8 fusion construct including DT386, (G4S)2 linker, and N‐terminal 6xHis tag. Fig. S2. SDS/PAGE analysis of DT386 expression and purification in E. coli. Fig. S3. SDS/PAGE analysis of DTCCL8 and DT386 proteins under reducing and non‐reducing conditions. Fig. S4. Cytotoxicity of DTCCL8 and unconjugated DT386 in BT549 and MDA‐MB‐231 cells. Fig. S5. Effect of DTCCL8 on immune cell populations and body weight in an independent in vivo study. [file MOL2-19-3376-s002.pdf]

Nucleotide Sequence (1452 pb)
NdeI – HisTag – DT386 – $(Gly_4Ser)_2$ – hCCL8 – HindIII
catatgcaccatcaccaccaccatggagctgatgacgtggttgattctagcaaatcttttgtaatggaaaatttctcctcctaccatggcacgaagccgggttatgttgactcaatccagaaaggcatccaaaagccgaagagcggcacgcagggt
aactacgacgacgactggaagggcttctatagcaccgataacaagtacgatgctgcgggttactcggtggacaacgaaaacccgctgtccggcaaggcgggtggtgtcgtcaaagtcacctacccgggtctgaccaaggtgttggccctgaa
agtagacaatgcggaaaccatcaaaaaagaactgggtctgtcgttaaccgaaccgttgatggagcaagtgggtactgaggaattcatcaagcgctttggcgatggtgccagccgtgtggtcctgtcattgccattcgcagagggctcgtcgtccgt
tgagtatattaacaactgggaacaggcaaaagcgcttagcgtggagttagagatcaactttgaaacacgtggtaagcgtggacaggatgcaatgtatgaatacatggcacaagcgtgtgctggtaatagagtgcgtcgcagcgtgggtagctct
ctgagctgcattaacctggattgggatgttattcgtgataaaacaaagacgaagattgaatctctaaaggagcacggtccgatcaaaaataagatgagcgaatccccgaataagaccgtgtccgaagagaaggcgaaacaatacctggagga
gttccaccagaccgctttggagcacccggagctctctgagctgaaaaccgttaccggcaccaatccggtttttgcaggcgcgaattacgcggcgtgggcagtcaacgttgctcaggtgattgatagcgagactgcggacaacctggagaaaac
gaccgcggctctctccatcctgccgggcatcggcagcgttatgggtattgccgacggcgcggttcaccataataccgaagaaattgtggctcaaagcattgcgttgtctagcctgatggttgcgcaagcaattccactggttggcgaattggttgaca
tcggctttgccgcgtataacttcgtggagagcatcattaacctgttccaagttgtgcacaacagctataaccgccctgcatatagcccgggtcataaaaccggcggtggtggtagtggtggaggcggcagccagccggattctgtgagcatcccga
tcacctgctgctttaacgtgatcaatcgtaaaatcccgattcaacgtctggagagttacacgcgtattactaacatccagtgcccgaaggaggcggtgatcttcaaaacccagcgtggcaaagaagtttgtgctgacccgaaagaacgctgggttc
gcgactccatgaagcatctggaccagattttccagaatctgaaaccgtaatgaaagctt

Amino Acid Sequence 479 AA
HisTag – DT386 – $(Gly_4Ser)_2$ – hCCL8
MHHHHHHGADDVVDSSKSFVMENFSSYHGTKPGYVDSIQKGIQKPKSGTQGNYDDDWKGFYSTDNKYDAAGYSVDNENPLSGKAGGVVKVTYPGLTKVLALKVDNAETI
KKELGLSLTEPLMEQVGTEEFIKRFGDGASRVVLSLPFAEGSSSVEYINNWEQAKALSVELEINFETRGKRGQDAMYEYMAQACAGNRVRRSVGSSLSCINLDWDVIRDKTK
TKIESLKEHGPIKNKMSESPNKTVSEEKAKQYLEEFHQTALEHPELSELKTVTGTNPVFAGANYAAWAVNVAQVIDSETADNLEKTTAALSILPGIGSVMGIADGAVHHNTEEIV
AQSIALSSLMVAQAIPLVGELVDIGFAAYNFVESIINLFQVVHNSYNRPAYSPGHKTGGGGSGGGGSQPDSVSIPITCCFNVINRKIPIQRLESYTRITNIQCPKEAVIFKTQRGKE
VCADPKERWVRDSMKHLDQIFQNLKP

Figure S1. Annotated nucleotide and amino acid sequences of the DTCCL8 fusion construct including DT386, $(G_4S)_2$ linker, and N-terminal 6×His tag. The nucleotide sequence of the DTCCL8 fusion construct is shown with the corresponding amino acid sequence. The sequence includes the first 386 amino acids of the diphtheria toxin (DT) fused to human CCL8, connected by a $(G_4S)_2$ linker (four glycines and one serine residue). The N-terminal region contains a 6x His tag for purification purposes.

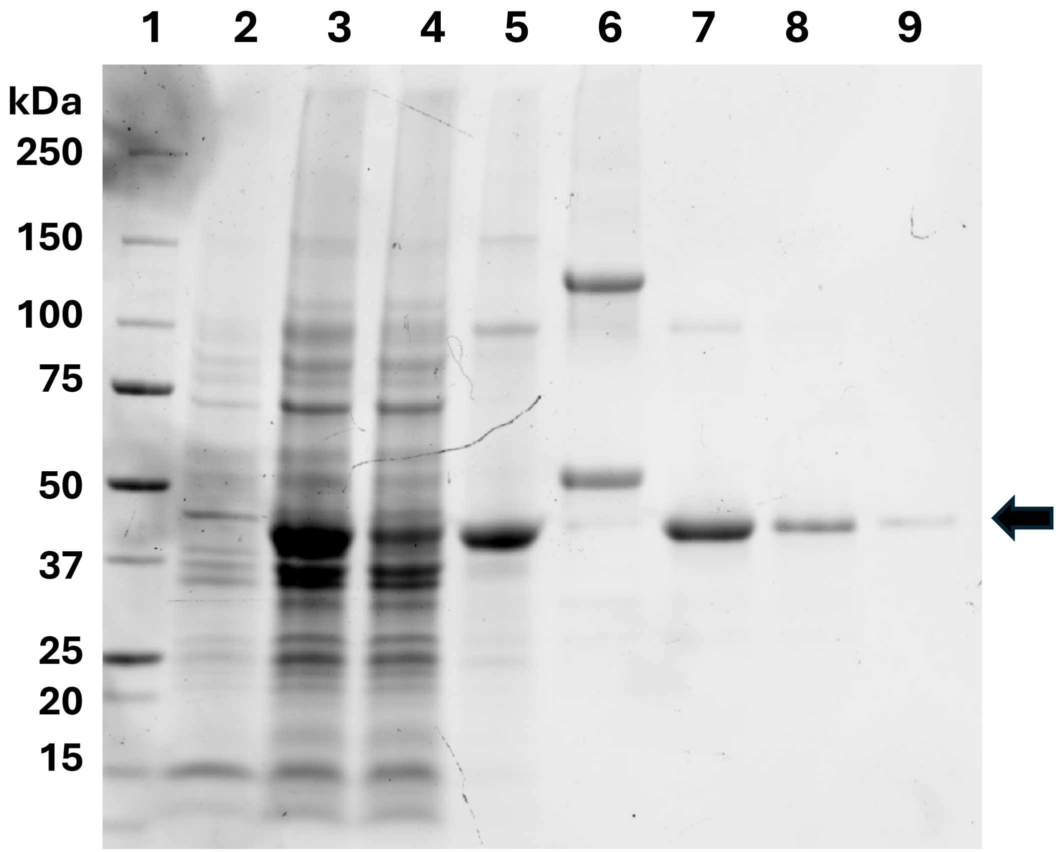

Figure S2. SDS-PAGE analysis of DT386 expression and purification in *E. coli.* SDS-PAGE analysis of transformed BL21 with pET30a-DT386, uninduced (lane 2), transformed BL21 with pET30a-DT386 induced with 0.3mM IPTG (lane 3), soluble proteins from lane 3 (lane 4), inclusion bodies isolated from lane 3 (lane 5), purified DTCCL8 (lane 6). Fractions of DT386 toxin purified by nickel affinity chromatography (lanes 7-9). Arrow indicates the predicted DT386 ~42.94 kDa band. Lane 1, molecular weight marker.

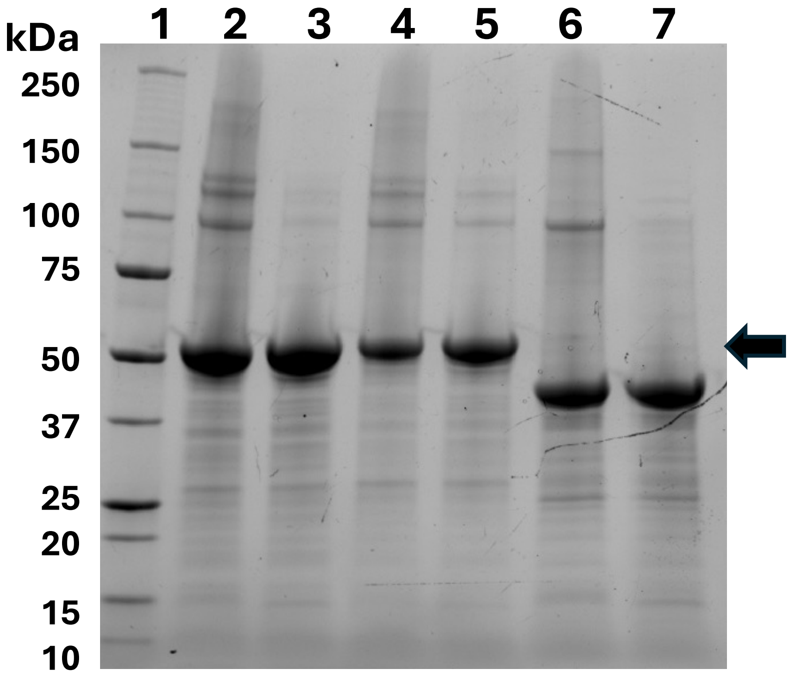

Figure S3. SDS-PAGE analysis of DTCCL8 and DT386 proteins under reducing and non-reducing conditions. Lane 1: molecular weight ladder. Lanes 2–3: DTCCL8 from the first batch, Lane 2 without DTT (non-reducing), Lane 3 with DTT (reducing). Lanes 4–5: DTCCL8 from the second batch, Lane 4 without DTT, Lane 5 with DTT. Lanes 6–7: DT386 protein, Lane 6 without DTT, Lane 7 with DTT. The expected molecular weight of DTCCL8 is 52.47 kDa, indicated by an arrow.

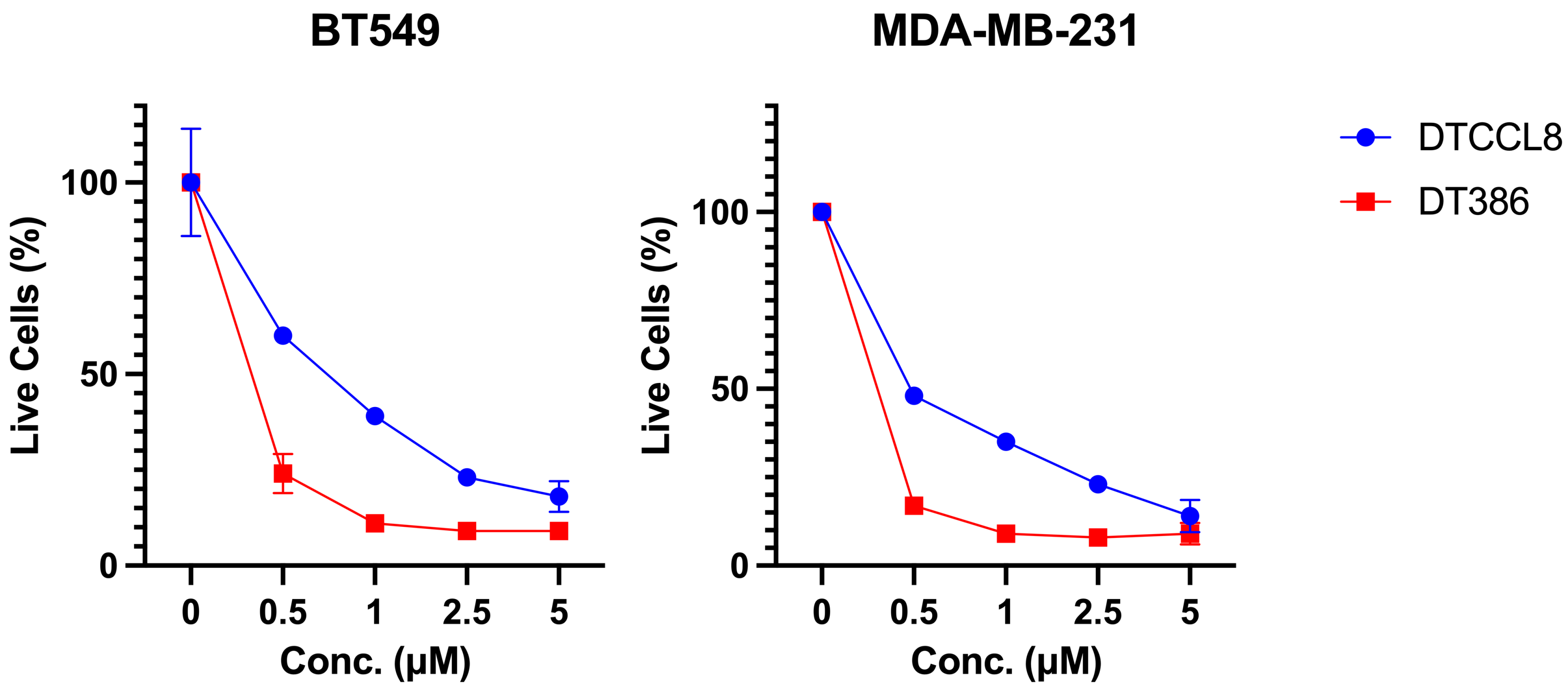

Figure S4. Cytotoxicity of DTCCL8 and unconjugated DT386 in BT549 and MDA-MB-231 cells. Cells were treated with DTCCL8 or DT386 at the indicated concentrations (Conc.; 0–5 μM) for 6 days. Cell viability was measured to assess toxin-induced cytotoxicity. DT386 shows consistently greater toxicity compared to DTCCL8 in both cell lines. Error bars represent mean ± SEM.

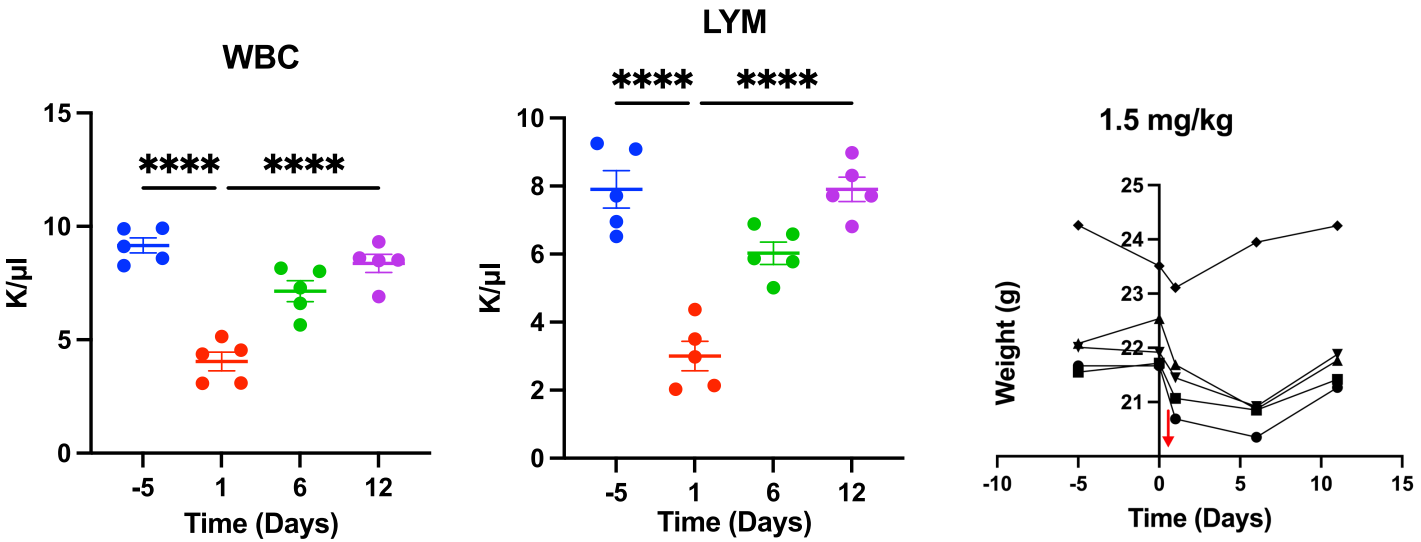

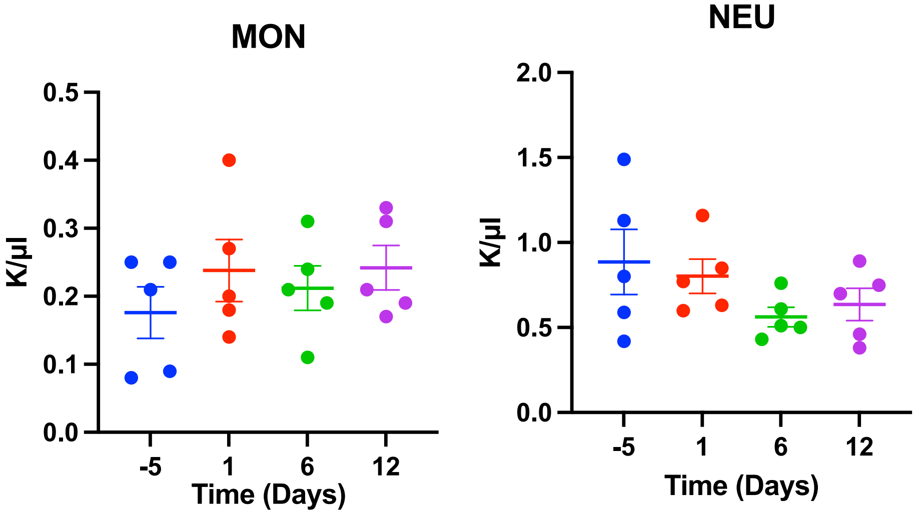

Figure S5. Effect of DTCCL8 on immune cell populations and body weight in an independent *in vivo* study. Mice were treated with DTCCL8 (1.5 mg/kg, intraperitoneally) at day 0. Blood counts were measured for total white blood cells (WBC), lymphocytes (LYM), monocytes (MON), and neutrophils (NEU) at the indicated time points. Body weight changes are shown in the right panel; each line represents an individual mouse. The red arrow marks the time of DTCCL8 administration. Error bars represent mean ± SEM. Statistical significance was determined using two-way ANOVA with Sidak's multiple comparisons test. ****$P < 0.0001$, ns=not significant.
