# Supplementary material for: Chimeric diphtheria toxin–CCL8 cytotoxic peptide for breast cancer management
Source: Mol Oncol. 2025 Jun 22;19(11):3376–86. doi: 10.1002/1878-0261.70079 (PMC12591328; doi:10.1002/1878-0261.70079)
Supplement: Supplementary file 2 — Video S1. Time‐lapse confocal imaging of DTCCL8 uptake in a representative HEK293T cell. [file MOL2-19-3376-s001.pptx]

## Slide 1
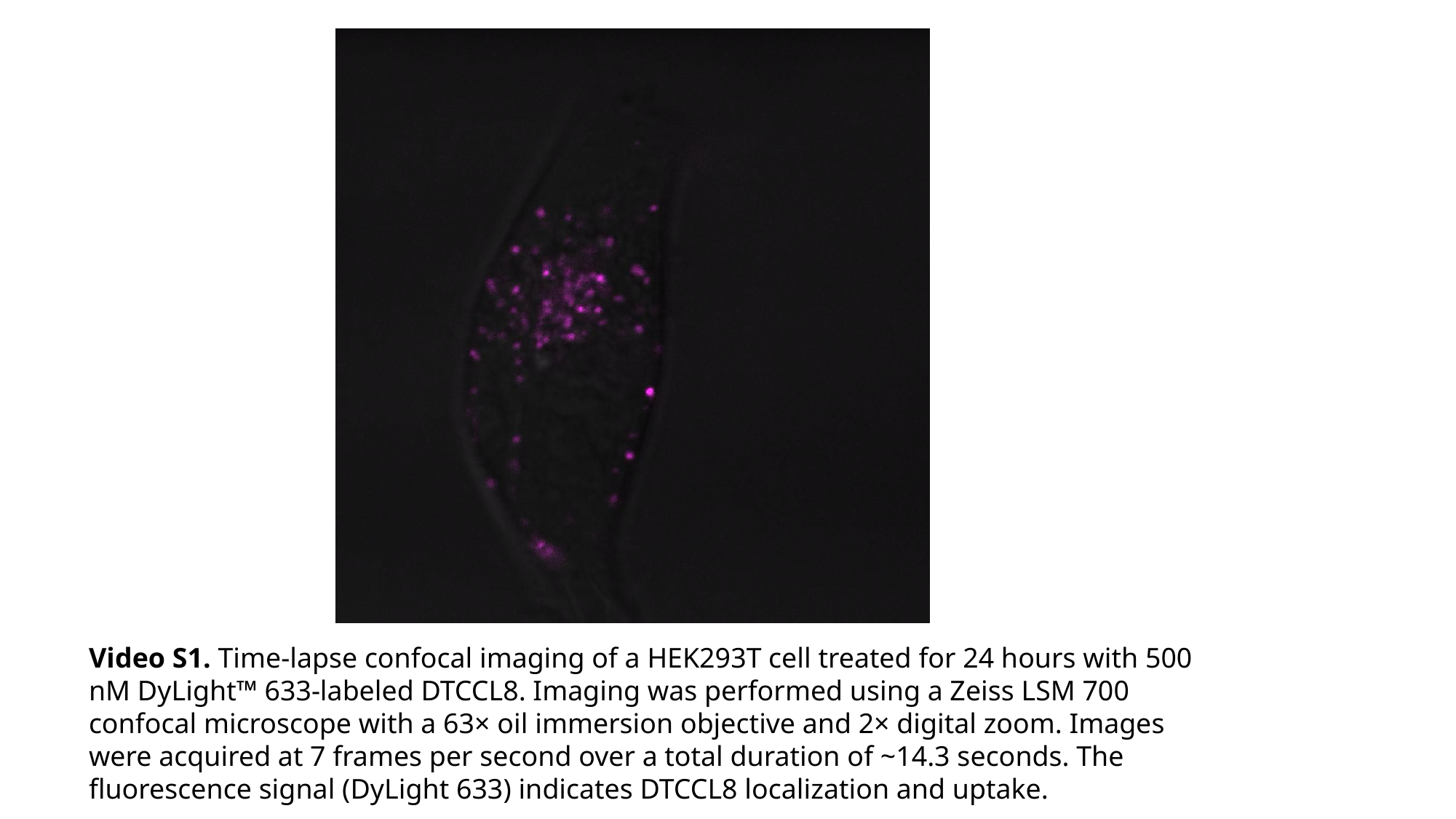

Video S1. Time-lapse confocal imaging of a HEK293T cell treated for 24 hours with 500 nM DyLight™ 633-labeled DTCCL8. Imaging was performed using a Zeiss LSM 700 confocal microscope with a 63× oil immersion objective and 2× digital zoom. Images were acquired at 7 frames per second over a total duration of ~14.3 seconds. The fluorescence signal (DyLight 633) indicates DTCCL8 localization and uptake.
